# Supplementary material for: Enhancing Prediction for Tumor Pathologic Response to Neoadjuvant Immunochemotherapy in Locally Advanced Esophageal Cancer by Dynamic Parameters from Clinical Assessments
Source: Cancers (Basel). 2023 Sep 1;15(17):4377. doi: 10.3390/cancers15174377 (PMC10486879; doi:10.3390/cancers15174377)

Table S1. ICC among the five parameters from esophagogram

	ICC	95%CI		p.value
Stenosis	0.794	0.611	0.897	<0.001
Dilation	0.753	0.542	0.875	<0.001
Shrinkage	0.775	0.581	0.886	<0.001
Smoothness	0.735	0.505	0.866	<0.001
Total Score	0.833	0.68	0.916	<0.001

Table S2. Multivariate logistic regression associating parameters from clinical assessments with pathological response

	pCR[a]				MPR[a]			
	OR	95% CI		p.value	OR	95% CI		p.value
Model1: CT+esophagogram								
GTV-post	0.978	0.922	1.035	0.449	0.954	0.906	1.002	0.059
post/pre	0.961	0.929	0.989	0.011	0.962	0.931	0.988	0.009
GTV-residual	0.998	0.992	1.004	0.599	1.000	0.995	1.005	0.921
Smoothness					2.609	1.682	4.310	<0.001
Esophagogram-total	1.209	1.089	1.362	0.001				
Dose reduction(above15%)	3.051	0.911	11.606	0.081				
Sex(male)	0.479	0.135	1.567	0.234				

Table S3. Clinical characteristics of patients in the testing and training cohorts

	testing N=41	trainning N=111	p.overall
NICE:			0.649
Others(q3w)	15 (36.6%)	47 (42.3%)	
NICE(qw)	26 (63.4%)	64 (57.7%)	
age	61.7 (8.00)	64.8 (7.11)	0.036
sex:			0.803
female	6 (14.6%)	20 (18.0%)	
male	35 (85.4%)	91 (82.0%)	
GTV-pre	53.6 (44.6)	36.2 (21.5)	0.021
tumor location:			0.613
LOWER	24 (58.5%)	55 (49.5%)	
MIDDLE	11 (26.8%)	37 (33.3%)	
UPPER	6 (14.6%)	19 (17.1%)	
clinical-T:			0.002
T1	0 (0.00%)	3 (2.70%)	
T2	11 (26.8%)	60 (54.1%)	
T3	30 (73.2%)	48 (43.2%)	
clinical-N:			0.264
N0	2 (4.88%)	4 (3.60%)	
N1	14 (34.1%)	22 (19.8%)	
N2	20 (48.8%)	69 (62.2%)	
N3	5 (12.2%)	16 (14.4%)	
MPR:			0.748
non-MPR	17 (41.5%)	41 (36.9%)	
MPR	24 (58.5%)	70 (63.1%)	
pCR:			0.898
non-pCR	27 (65.9%)	70 (63.1%)	
pCR	14 (34.1%)	41 (36.9%)	
TRG:			0.960
TRG1	14 (34.1%)	41 (36.9%)	
TRG2	10 (24.4%)	29 (26.1%)	
TRG3	5 (12.2%)	11 (9.91%)	
TRG4	12 (29.3%)	30 (27.0%)	
yp-T:			0.690
T0	14 (34.1%)	41 (36.9%)	
T1	6 (14.6%)	22 (19.8%)	
T2	7 (17.1%)	12 (10.8%)	
T3	14 (34.1%)	36 (32.4%)	
yp-N:			0.209
N0	27 (65.9%)	59 (53.2%)	
N1	11 (26.8%)	27 (24.3%)	
N2	2 (4.88%)	18 (16.2%)	
N3	1 (2.44%)	7 (6.31%)	

Figure S1. GTV-pre and GTV-PET-pre agreement analysis by Passing-Bablok regression

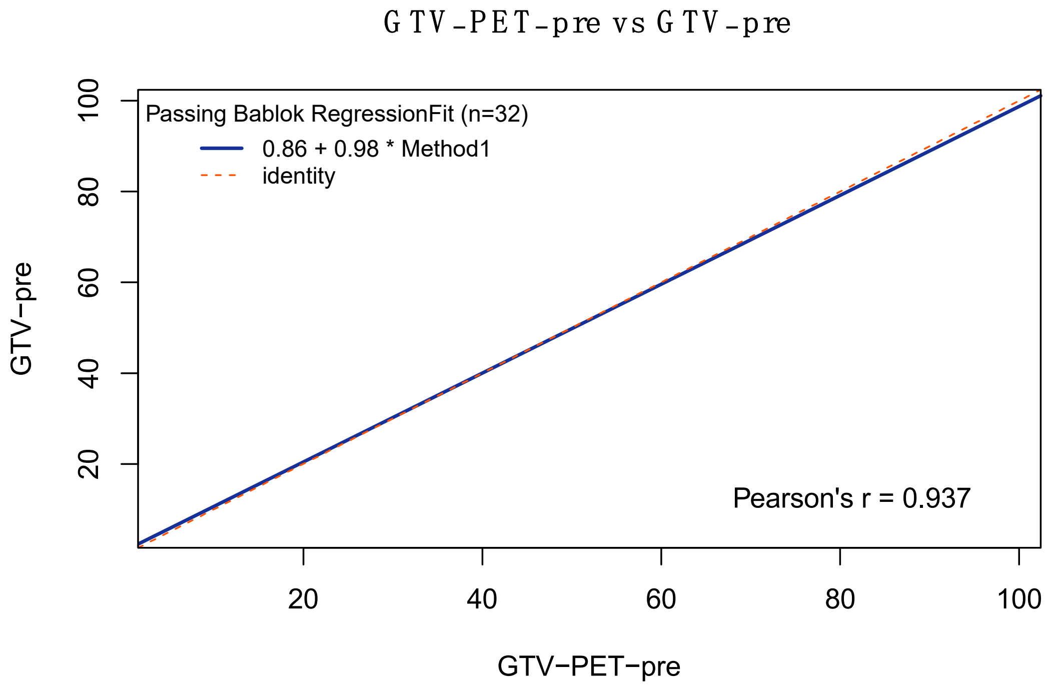

Figure S2. (A) The calibration plot of the MPR Model 2 in the testing cohort. (B) The decision curve for the testing cohort of the MPR Model 2 nomogram.

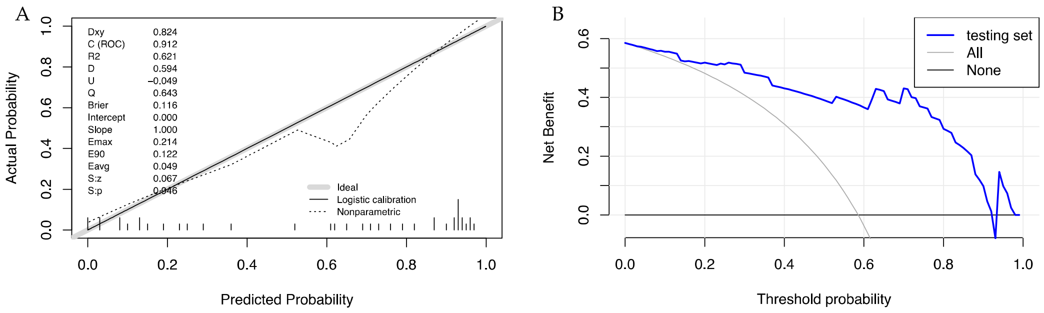

Supplement: Supplementary file 1 [file cancers-15-04377-s001.zip › cancers-2536773-supplementary.pdf]
